# Supplementary material for: Increases in multiple resources promote competitive ability of naturalized non-native plants
Source: Commun Biol. 2022 Oct 30;5:1150. doi: 10.1038/s42003-022-04113-1 (PMC9618556; doi:10.1038/s42003-022-04113-1)
Supplement: Supplementary file 2 — Supplementary Information [file 42003_2022_4113_MOESM2_ESM.pdf]

Increases in multiple resources promote competitive ability of naturalized non-native plants

Zhijie Zhang, Yanjie Liu, Angelina Hardrath, Huifei Jin, Mark van Kleunen

Supplementary information

Supplement Note 1 Effects of nutrient and light availabilities

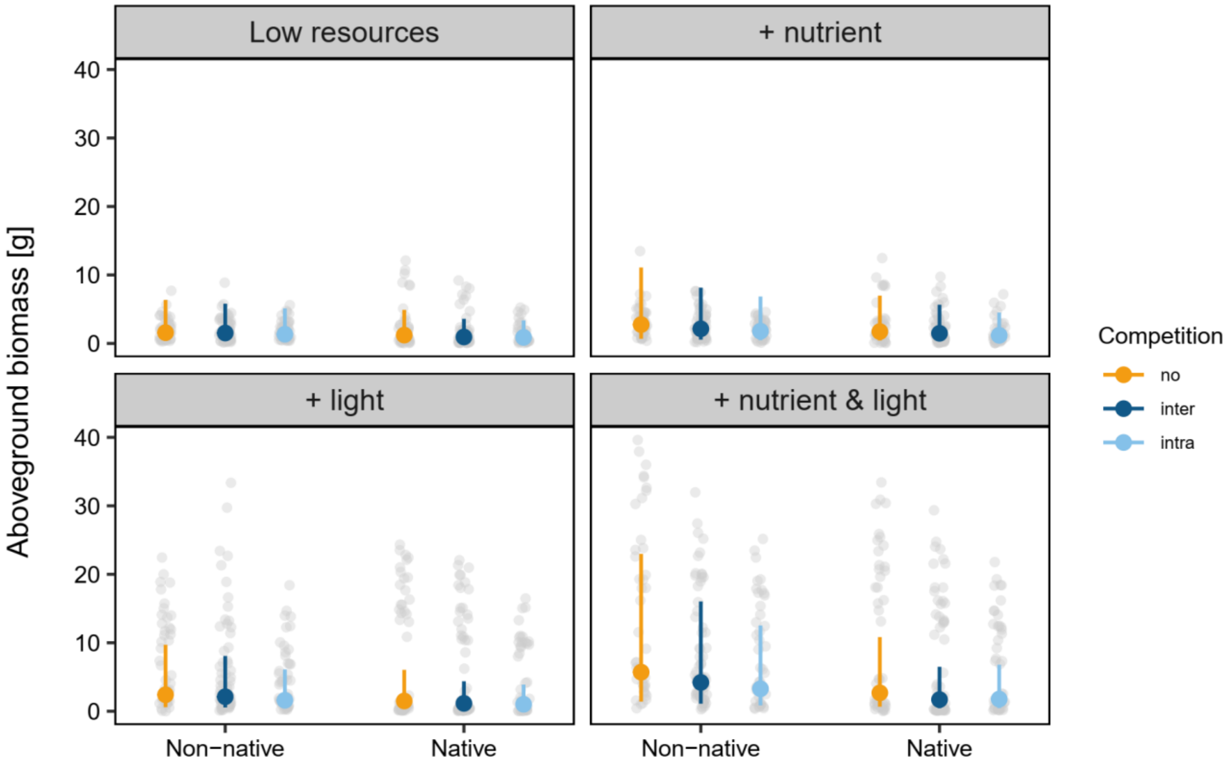

Supplementary Figure 4 Effects of nutrient and light availabilities on aboveground biomass of native and non-native plants. Addition of nutrients or light significantly increased the effect of competition (plants grown alone vs. plants with competition). However, this effect did not significantly depend on origin of the plants (i.e. there was no significant effect of origin: $Comp_{without\text{-}with}$: nutr, origin: $Comp_{without\text{-}with}$: light or origin: $Comp_{without\text{-}with}$: nutr: light in **Supplementary Table 2**). The effect of intraspecific competition was overall stronger than the effect of interspecific competition, and this effect did not significantly depend on origin of plants or resource treatments. Grey dots indicate the raw data.

Supplement Note 2 Separate analyses of the two experiments

Statistical analyses

The experiment in China

The statistical model was similar to the one that we used in the joint analysis of both experiments. In brief, we included aboveground biomass as response variable; origin of the species (non-native or native), competition treatment, nutrient treatment, light treatment and their interactions as fixed effects; and identity and family of the species as random effects. In addition, we added log-transformed initial height as a covariate. We allowed each species to respond differently to the nutrient and light treatments (i.e. we included random slopes). However, in this model, the competition treatment had four instead of three levels: 1) competition-free, 2) intraspecific competition, 3) interspecific competition from non-native species, and 4) interspecific competition from native species. We created three variables to split the competition treatment into three contrasts to test 1) the effect of competition, 2) the difference between intra- and inter-specific competition, and 3) the difference between interspecific competition from non-native species and that from native species.

The experiment in Germany

The model was the same as for the experiment in China. However, like in the joint analysis, the competition treatment had only three levels: 1) competition-free, 2) intraspecific competition, and 3) interspecific competition between native and non-native species. Therefore, we broke down the competition treatments into separate contrasts using the same variables as in the joint analysis. In addition, because cage effect is identical to the light treatment, it was not added as a random block effect to avoid overfitting.

Results

Consistent with the joint analysis, the individual analyses of each experiment separately showed that biomass production of plants increased with increases of nutrients and light intensity (Supplementary Figure 1a&b; Supplementary Table 2). In addition, both experiments found that the competitive outcome between natives and non-natives was affected by the interaction between the nutrient and light treatments (Supplementary Figure 1a&b; Supplementary Table 2). More specifically, under low resource availabilities or with addition of only one type of resource, biomass production did not differ between natives and non-natives, whereas with a joint increase of nutrients and light, non-natives produced more biomass than natives.

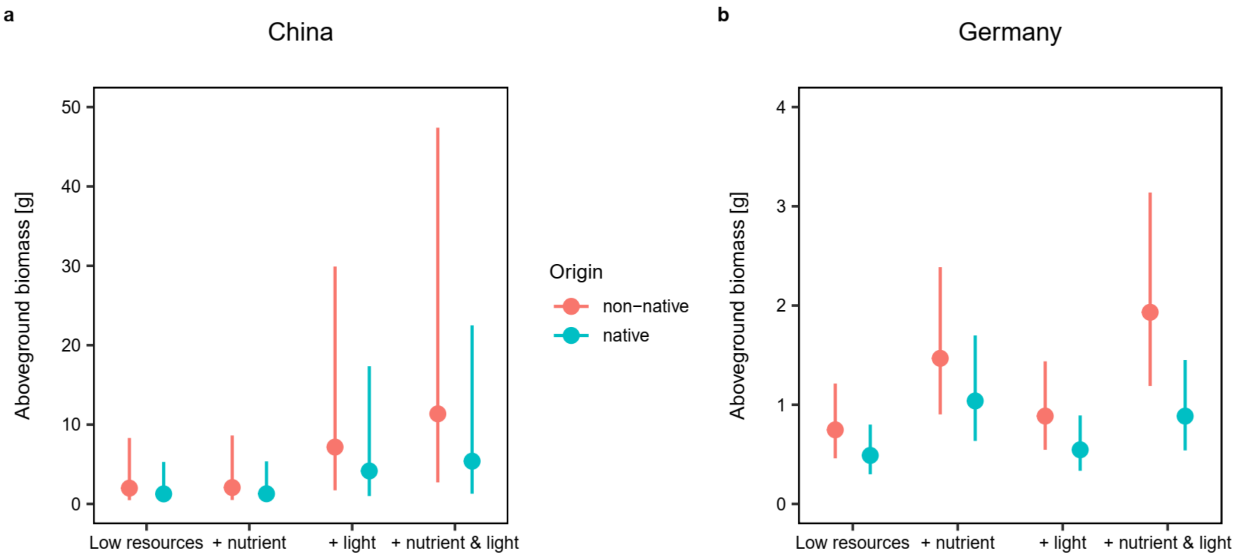

Supplementary Figure 1 Effects of nutrient and light availabilities on competitive outcomes between non-native (red) and native (blue) plants. Data from the experiments in China (a) and Germany (b) were analyzed separately Competitive outcome is indicated by the difference in average biomass production. For example, a higher biomass production of non-native plants indicates that non-natives will outcompete natives. Error bars indicate 95% CIs.

Consistent with the joint analysis, the experiment in China showed that biomass production largely increased with a joint increase of nutrients and light, as indicated by the interaction between nutrient and light treatments (Supplementary Figure 1a; Supplementary Table 2). Furthermore, this experiment included competition between two non-natives and that between two natives. It showed that the origin of competitor species also matters (Supplementary Figure 2): non-natives produced more biomass when competing with natives than when competing with other non-natives, whereas biomass production of natives was not strongly affected by the origin of the competitor species.

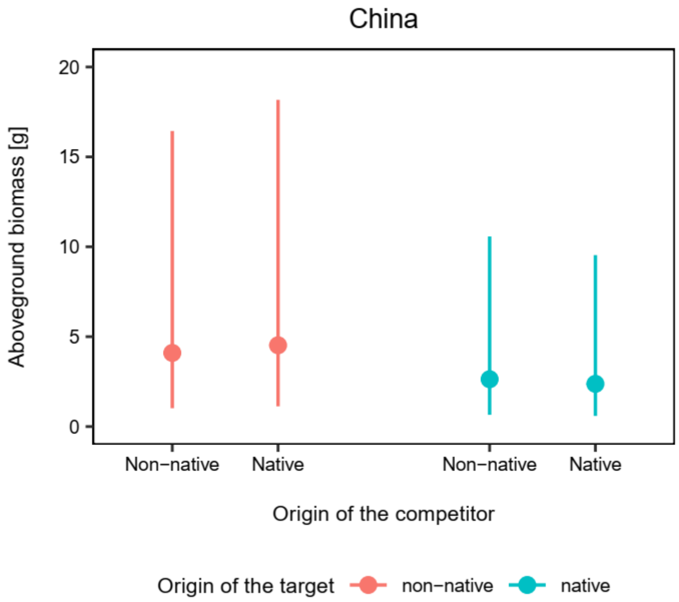

Supplementary Figure 2 Competitive outcomes among native and non-native plants for the experiment in China. Red and blue colors indicate non-native and native species, respectively, which were grown with interspecific competitors. The competitor was either a non-native species or a native one. Error bars indicate 95% CIs.

The experiment in Germany showed that the competitive outcome between native and non-native species was affected by light intensity (Supplementary Figure 1b). Under low light intensity, biomass production did not strongly differ between natives and non-natives, whereas non-natives produced more biomass than natives with an increase of light intensity. This effect is mainly driven by the fact that non-natives produced more biomass than natives with a joint increase of nutrients and light intensity (Supplementary Figure 1b), and by the fact that non-natives produced more biomass than natives under interspecific competition and high light-intensity (Supplementary Figure 3)

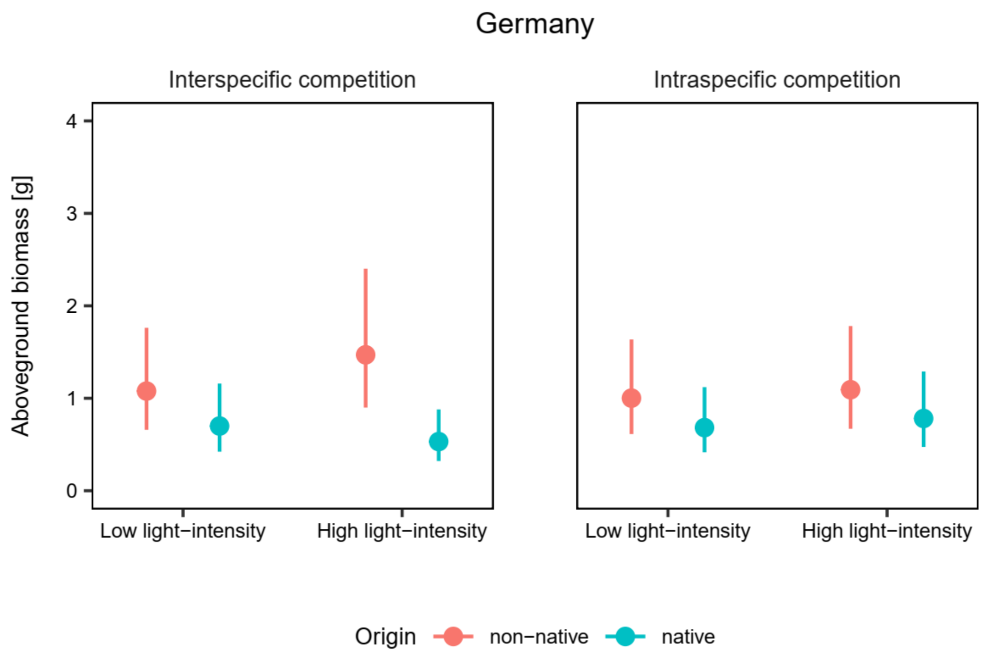

Supplementary Figure 3 Effect of light availability on non-native (red) and native (blue) plants under inter- or intraspecific competition. Error bars indicate 95% CIs.

Supplement Note 3 Calculation of competition-contrast variables.

To set two contrast variables to test 1) the effect of the presence of competitors, and 2) the difference between intra- and interspecific competition. We set the initial contrast matrix (Mat) as:

Competition treatments	Intercept	No competition vs competition	Intra- vs interspecific
No competition	1/3	-1	0
Intraspecific competition	1/3	1/2	1
Interspecific competition	1/3	1/2	-1

This initial contrast matrix assumes that 1) the biomass in the no-competition treatment equals the average of the biomass in the intra- and interspecific competition treatments, and that 2) biomass in the intraspecific competition equals the biomass in the interspecific competition treatment.

The final contrast matrix (Mat_final) is the inverse of the initial contrast matrix (Mat) transposed, which results in:

Competition treatments	Intercept	No competition vs competition	Intra- vs interspecific
No competition	1	-2/3	0
Intraspecific competition	1	1/3	1/2
Interspecific competition	1	1/3	-1/2

The two final contrast variables are the ‘No competition vs competition’ and ‘Intra- vs interspecific’ variables of the final contrast matrix.

Supplementary Table 1 The 24 herbaceous species used in the experiment in China and the experiment in Germany

**Species**	**Site**	**Family**	**Origin**	**Propagule**	**Sowing date**
Alternanthera philoxeroides	China	Amaranthaceae	non-native	stem fragment	24 June 2020
Alternanthera sessilis	China	Amaranthaceae	native	stem fragment	23 June 2020
Bidens pilosa	China	Compositae	non-native	seed	27 June 2020
Solidago canadensis	China	Compositae	non-native	seed	21 May 2020
Bidens maximowicziana	China	Compositae	native	seed	27 June 2020
Solidago decurrens	China	Compositae	native	seed	21 May 2020
Paspalum notatum	China	Poaceae	non-native	seed	24 June 2020
Paspalum orbiculare	China	Poaceae	native	stem fragment	24 June 2020
Diplotaxis muralis	Germany	Brassicaceae	non-native	seed	15 June 2020
Cardamine hirsuta	Germany	Brassicaceae	native	seed	15 June 2020
Galinsoga parviflora	Germany	Compositae	non-native	seed	15 June 2020
Hieracium pilosella	Germany	Compositae	native	seed	15 June 2020
Elsholtzia ciliata	Germany	Lamiaceae	non-native	seed	15 June 2020
Salvia verticillata	Germany	Lamiaceae	non-native	seed	15 June 2020
Mentha longifolia	Germany	Lamiaceae	native	seed	15 June 2020
Prunella vulgaris	Germany	Lamiaceae	native	seed	15 June 2020
Onobrychis viciifolia	Germany	Leguminosae	non-native	seed	15 June 2020
Vicia villosa	Germany	Leguminosae	non-native	seed	15 June 2020
Medicago falcata	Germany	Leguminosae	native	seed	15 June 2020
Medicago lupulina	Germany	Leguminosae	native	seed	15 June 2020
Epilobium ciliatum	Germany	Onagraceae	non-native	seed	15 June 2020
Epilobium hirsutum	Germany	Onagraceae	native	seed	15 June 2020
Eragrostis minor	Germany	Poaceae	non-native	seed	15 June 2020
Vulpia myuros	Germany	Poaceae	native	seed	15 June 2020

continued:

**Species**	**Propagule source**	**Distribution**[1]	**Life form**[2]	**Function group**
Alternanthera philoxeroides	wild population	17	long	forb
Alternanthera sessilis	wild population	70	long	forb
Bidens pilosa	wild population	34	short	forb
Solidago canadensis	wild population	67	long	forb
Bidens maximowicziana	wild population	10	short	forb
Solidago decurrens	wild population	20	long	forb
Paspalum notatum	commercial	8	long	grass
Paspalum orbiculare	wild population	28	long	grass
Diplotaxis muralis	Uni-Konstanz[3]	37	short	forb
Cardamine hirsuta	Uni-Konstanz	89	short	forb
Galinsoga parviflora	commercial	30	short	forb
Hieracium pilosella	commercial	37	long	forb
Elsholtzia ciliata	Uni-Konstanz	34	short	forb
Salvia verticillata	commercial	28	long	forb
Mentha longifolia	commercial	74	long	forb
Prunella vulgaris	commercial	164	long	forb
Onobrychis viciifolia	commercial	9	long	forb
Vicia villosa	commercial	47	long	forb
Medicago falcata	commercial	57	long	forb
Medicago lupulina	commercial	72	long	forb
Epilobium ciliatum	Uni-Konstanz	51	long	forb
Epilobium hirsutum	Uni-Konstanz	95	long	forb
Eragrostis minor	commercial	87	short	grass
Vulpia myuros	commercial	65	short	grass

[1]Native distribution in terms of number of TDWG3 regions in the POWO database (https://powo.science.kew.org/)
[2]Annual or biennial plant are classified as short-lived plants, and perennial as long-lived.
[3]Botanical Garden of the University of Konstanz

Supplementary Table 2 Effects of the origin of target species, competition treatments and resource availabilities on aboveground biomass of plants for each of the experiments (China, Germany) separately and for the experiments jointly.

	China				Germany				Joint			
Items	DF	χ^2	p-value		DF	χ^2	p-value		DF	χ^2	p-value	
log(initial height)	1	68.819	<0.001		-	-	-		-	-	-	
origin	1	0.301	0.613		1	3.637	0.057	†	1	2.042	0.153	
Comp$_{\text{without-with}}$[1]	1	3.313	0.067	†	1	1.976	0.16		1	2.087	0.149	
Comp$_{\text{intra-inter}}$[1]	1	20.281	<0.001	*	1	0.199	0.655		1	20.054	<0.001	*
Comp$_{\text{native-non-native}}$[2]	1	0.022	0.990		-	-	-		-	-	-	
nutrients (nutr)	1	5.55	<0.001	*	1	99.935	<0.001	*	1	46.424	<0.001	*
light	1	44.059	<0.001	*	1	5.626	0.018	*	1	3.176	0.075	†
origin:Comp$_{\text{without-with}}$	1	0.146	0.668		1	1.152	0.283		1	0.626	0.429	
origin: Comp$_{\text{intra-inter}}$	1	0.833	0.461		1	2.588	0.108	†	1	0.355	0.551	
origin: Comp$_{\text{native-non-native}}$	1	8.761	0.015	*	-	-	-		-	-	-	
origin: nutr	1	0.434	0.822		1	0.442	0.506		1	0.817	0.366	
Comp$_{\text{without-with}}$: nutr	1	0.188	0.597		1	21.74	<0.001	*	1	5.209	0.022	*
Comp$_{\text{intra-inter}}$: nutr	1	0.285	0.328		1	0.86	0.354		1	0.002	0.96	
Comp$_{\text{native-non-native}}$: nutr	1	0.141	0.930		-	-	-		-	-	-	
origin: light	1	0.183	0.673		1	4.698	0.03	*	1	1.602	0.206	
Comp$_{\text{without-with}}$: light	1	2.718	0.077		1	1.674	0.196		1	4.214	0.040	*
Comp$_{\text{intra-inter}}$: light	1	0.107	0.858		1	0.207	0.649		1	0.021	0.884	
Comp$_{\text{native-non-native}}$: light	1	<0.001	0.949		-	-	-		-	-	-	
nutr: light	1	43.581	<0.001	*	1	0.026	0.872		1	24.122	<0.001	*
origin: Comp$_{\text{without-with}}$: nutr	1	0.409	0.886		1	0.013	0.911		1	1.91	0.167	
origin: Comp$_{\text{intra-inter}}$: nutr	1	0.845	0.190		1	1.123	0.289		1	0.011	0.918	
origin: Comp$_{\text{native-non-native}}$: nutr	1	0.205	0.946		-	-	-		-	-	-	
origin: Comp$_{\text{without-with}}$: light	1	0.245	0.870		1	0.28	0.596		1	0.061	0.805	
origin: Comp$_{\text{intra-inter}}$: light	1	0.681	0.607		1	7.099	0.008	*	1	3.065	0.080	†
origin: Comp$_{\text{native-non-native}}$: light	1	0.419	0.341		-	-	-		-	-	-	
origin: nutr: light	1	3.435	0.061	†	1	3.839	0.050	*	1	4.655	0.031	*

Continued:

	China				Germany				Pooled		
items	DF	χ^2	p-value		DF	χ^2	p-value		DF	χ^2	p-value
Comp$_{without-with}$: nutr: light	1	0.566	0.315		1	0.906	0.341		1	0.021	0.884
Comp$_{intra-inter}$: nutr: light	1	3.003	0.052	†	1	1.421	0.233		1	2.412	0.120
Comp$_{native-non-native}$: nutr: light	1	0.759	0.417		-	-	-		-	-	-
origin: Comp$_{without-with}$: nutr: light	1	0.168	0.792		1	3.237	0.072	†	1	0.871	0.351
origin: Comp$_{intra-inter}$: nutr: light	1	0.313	0.541		1	1.611	0.204		1	0.932	0.334
origin: Comp$_{native-non-native}$: nutr: light	1	1.627	0.205		-	-	-		-	-	-
**Random effects**	SD				SD				SD		
Study site	-				-				0.843		
Family	0.006				0.286				0.178		
Species	1.311				0.509				0.844		
Family of the competitor	<0.001				0.112				0.001		
Competitor species	0.145				0.176				0.204		
light:species	0.413				0.070				0.302		
nutr:species	0.130				0.125				0.217		
cage_first[3]	-				<0.001				0.284		
cage_second[3]	-				<0.001				<0.001		
pot	0.041				<0.001				<0.001		
Residual	0.360				0.726				0.346		

[1]The competition treatments were split into contrasts to test the effect of competition (Comp$_{without-with}$) and the difference between intra- and interspecific competition (Comp$_{intra-inter}$).

[2]For the experiment in China, which included competition between two natives and between two non-natives, there was an additional contrast to test for a difference between interspecific competition from non-natives and that from natives (Comp$_{native-non-native}$).

[3]For the experiment in Germany, pots were randomized twice across cages, so that there were two ‘cage’ (i.e. block) effects.
