# Supplementary material for: Increases in multiple resources promote competitive ability of naturalized non-native plants
Source: Commun Biol. 2022 Oct 30;5:1150. doi: 10.1038/s42003-022-04113-1 (PMC9618556; doi:10.1038/s42003-022-04113-1)
Supplement: Supplementary file 3 — Reporting Summary-New [file 42003_2022_4113_MOESM3_ESM.pdf]

nature portfolio

Corresponding author(s): Yanjie Liu

Last updated by author(s): Oct 4, 2022

Reporting Summary

Nature Portfolio wishes to improve the reproducibility of the work that we publish. This form provides structure for consistency and transparency in reporting. For further information on Nature Portfolio policies, see our Editorial Policies and the Editorial Policy Checklist.

Statistics

For all statistical analyses, confirm that the following items are present in the figure legend, table legend, main text, or Methods section.

n/a	Confirmed	
☐	☒	The exact sample size (*n*) for each experimental group/condition, given as a discrete number and unit of measurement
☐	☒	A statement on whether measurements were taken from distinct samples or whether the same sample was measured repeatedly
☒	☐	The statistical test(s) used AND whether they are one- or two-sided<br>*Only common tests should be described solely by name; describe more complex techniques in the Methods section.*
☐	☒	A description of all covariates tested
☐	☒	A description of any assumptions or corrections, such as tests of normality and adjustment for multiple comparisons
☐	☒	A full description of the statistical parameters including central tendency (e.g. means) or other basic estimates (e.g. regression coefficient) AND variation (e.g. standard deviation) or associated estimates of uncertainty (e.g. confidence intervals)
☐	☒	For null hypothesis testing, the test statistic (e.g. *F*, *t*, *r*) with confidence intervals, effect sizes, degrees of freedom and *P* value noted<br>*Give P values as exact values whenever suitable.*
☒	☐	For Bayesian analysis, information on the choice of priors and Markov chain Monte Carlo settings
☐	☒	For hierarchical and complex designs, identification of the appropriate level for tests and full reporting of outcomes
☒	☐	Estimates of effect sizes (e.g. Cohen's *d*, Pearson's *r*), indicating how they were calculated

Our web collection on statistics for biologists contains articles on many of the points above.

Software and code

Policy information about availability of computer code

Data collection	All data were collected from two greenhouse experiments, one in Germany, and another in China.
Data analysis	All analyses were performed using R version 3.6.1.

For manuscripts utilizing custom algorithms or software that are central to the research but not yet described in published literature, software must be made available to editors and reviewers. We strongly encourage code deposition in a community repository (e.g. GitHub). See the Nature Portfolio guidelines for submitting code & software for further information.

Data

Policy information about availability of data

All manuscripts must include a data availability statement. This statement should provide the following information, where applicable:

- Accession codes, unique identifiers, or web links for publicly available datasets
- A description of any restrictions on data availability
- For clinical datasets or third party data, please ensure that the statement adheres to our policy

Should the manuscript be accepted, the data and code of the study will be archived in Figshare and the DOI will be included at the end of the article.

Human research participants

Policy information about studies involving human research participants and Sex and Gender in Research.

Reporting on sex and gender	n/a
Population characteristics	n/a
Recruitment	n/a
Ethics oversight	n/a

Note that full information on the approval of the study protocol must also be provided in the manuscript.

Field-specific reporting

Please select the one below that is the best fit for your research. If you are not sure, read the appropriate sections before making your selection.

☐ Life sciences ☐ Behavioural & social sciences ☒ Ecological, evolutionary & environmental sciences

For a reference copy of the document with all sections, see nature.com/documents/nr-reporting-summary-flat.pdf

Ecological, evolutionary & environmental sciences study design

All studies must disclose on these points even when the disclosure is negative.

Study description	With two similar common garden experiments, one in China and one in Germany, we tested whether nutrient and light availabilities affected the competitive outcomes, in terms of biomass production, between naturalized alien and native plants.
Research sample	For the experiment in China, we selected eight species that are either native or alien in China.<br>For the experiment in Germany, we selected 16 species that are either native or alien in Germany.
Sampling strategy	For the experiment in China, we grew all eight species without competition, in intraspecific competition and in all 28 possible pairs of interspecific competition. For the competition-free and intraspecific-competition treatments, we replicated each species seven times (i.e. we had seven technical replicates). For the interspecific-competition treatment, for which we had many pairs of species (i.e. biological replicates), we replicated each pair two times.<br>For the experiment in Germany, we imposed three competition treatments: competition-free, intraspecific competition and interspecific competition. However, in this experiment, which had two times more species than the experiment in China, we only included 24 randomly chosen species pairs for the interspecific-competition treatment, and all of these pairs consisted of one naturalized alien and one native species. For the competition-free treatment, we replicated each species two times (i.e. we had two technical replicates). For the competition treatments, we did not use technical replicates for any of the species combinations for logistic reasons. However, as we had a large number of species pairs in the inter-specific competition treatment, we had many biological replicates.
Data collection	After the end of the experiments, we harvested the aboveground biomass of all plants.
Timing and spatial scale	The experiment in China started from 21 May 2020 to 1 September 2020.<br>The experiment in Germany started from On 15 June 2020 to 8 September 2020.
Data exclusions	No data were excluded from the analysis.
Reproducibility	The reproducibility can be verified by each other of the two experiments.
Randomization	All pots were randomly assigned, and re-randomized once during the experiment.
Blinding	Blinding was not applicable for the plant study.

Did the study involve field work? ☐ Yes ☒ No

Reporting for specific materials, systems and methods

We require information from authors about some types of materials, experimental systems and methods used in many studies. Here, indicate whether each material, system or method listed is relevant to your study. If you are not sure if a list item applies to your research, read the appropriate section before selecting a response.

Materials & experimental systems

n/a	Involved in the study
☒	☐ Antibodies
☒	☐ Eukaryotic cell lines
☒	☐ Palaeontology and archaeology
☒	☐ Animals and other organisms
☒	☐ Clinical data
☒	☐ Dual use research of concern

Methods

n/a	Involved in the study
☒	☐ ChIP-seq
☒	☐ Flow cytometry
☒	☐ MRI-based neuroimaging
